# Adhesion Properties Between Rubber Asphalt Mastic and Aggregate: Verification from Surface Free Energy Theory and Molecular Dynamics

**DOI:** 10.3390/ma18133115

**Published:** 2025-07-01

**Authors:** Huajia Yin, Shenyang Cao, Fucheng Guo, Xu Wu

**Affiliations:** 1 Gansu Jiayuguan Highway Development Center, Jiayuguan 735100, China; yhj18909474361@hotmail.com; 2 Gansu New Development Investment Group Ltd., Lanzhou 730030, China; 3 School of Civil Engineering, Lanzhou University of Science and Technology, Lanzhou 730050, China; 4 School of Civil Engineering, Lanzhou Jiaotong University, Lanzhou 730070, China; wx18893105922@hotmail.com

**Keywords:** rubber asphalt mastic, aggregate, surface free energy, molecular dynamics, adhesion properties

## Abstract

The adhesive properties between rubber asphalt mastic and aggregate are crucial to rubber asphalt mixtures’ stability and moisture resistance. This paper employs surface free energy (SFE) theory and molecular dynamics (MD) to examine the bond strength and debonding behavior at the rubber asphalt mastic–aggregate interface. The results showed that the dispersion fraction of RC1.0 was 7.12 mJ/m^2^ higher than that of RA, and the limestone mineral powder improved the adhesion properties of rubberized asphalt to aggregate and the anti-stripping properties. SiO_2_ and CaCO_3_ are contributors to the van der Waals and electrostatic forces between rubber asphalt–aggregate, respectively. The high concentration of mineral powder has a bridging effect in rubber asphalt mastic–aggregate. CaCO_3_ filler is more pronounced in enhancing the adhesion properties of rubber asphalt–aggregate. CaCO_3_ mineral powder mainly improves the anti-debonding ability of rubber asphalt–aggregate by reducing the thickness of water film between rubber asphalt–aggregate.

## 1. Introduction

Asphalt pavement undergoes prolonged exposure to environmental factors and traffic loads, leading to various forms of deterioration that significantly reduce its service life [1,2,3]. Consequently, there is a critical demand for road construction materials that combine high-temperature resistance to permanent deformation with favorable fatigue performance at moderate temperatures. To meet these requirements, numerous asphalt modifiers have been developed and applied [4,5,6]. Research demonstrates that incorporating an optimal amount of crumb rubber from recycled tires not only fulfills these functional objectives but also offers an environmentally sustainable solution for tire waste management [7,8,9,10].

The adhesion properties between asphalt and aggregate play a critical role in determining the moisture stability and long-term durability of asphalt mixtures. Recently, the interfacial bonding behavior of rubberized asphalt and aggregates has gained significant research attention. Guo [11] employed a molecular dynamics approach to analyze the influence of rubber content and aggregate type on the adhesive characteristics of the rubberized asphalt–aggregate interface. Xie et al. [12] demonstrated that styrene-butadiene rubber (SBR) enhances van der Waals forces at the interface, thereby improving bond strength. Based on surface free energy theory, Li [13] found that rubber modification increases the asphalt’s surface energy, dispersive component, and adhesion work, further enhancing its bonding performance. Additionally, Zhang [14], Jiao [15], Chen [16], and Mohamed et al. [17] have contributed to the exploration of adhesion mechanisms in rubberized asphalt–aggregate systems.

Asphalt mastic, a blend of asphalt binder and mineral filler, is a vital component in asphalt mixtures. Studies show that mixture failures often initiate at the mastic–aggregate interface, making interfacial adhesion crucial for moisture resistance and overall durability. Xu [18] demonstrated that SiO_2_ nanoparticles enhance bonding by strengthening electrostatic interactions at the interface. Tan [19] used surface free energy theory to systematically assess adhesion between mastic and aggregate, while Zhang [20] employed pull-off tests to study how filler composition affects interfacial bonding. However, research on rubber-modified asphalt mastic remains limited, and conventional testing methods often fall short in explaining the complex adhesion mechanisms in rubberized mastic–aggregate systems.

**Figure 1:**
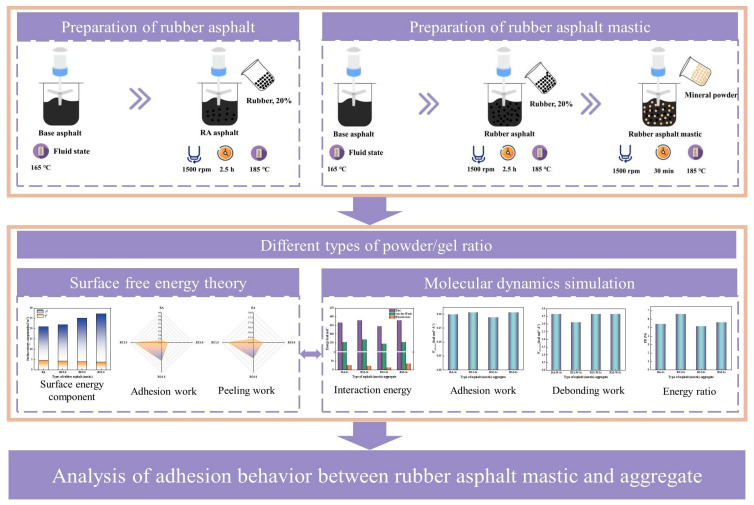
Technical flow chart.

The existing studies mostly focus on the properties of rubber-modified asphalt itself or the ordinary asphalt–aggregate interface, whereas the present study combines the two and focuses on the composite interface system of rubber-modified asphalt mortar (with mineral filler) and aggregate. This study investigates the synergistic effect of two variables, “mineral filler” and “aggregate type”, on the interfacial adhesion of rubber-modified asphalt and reveals the adhesion mechanism of the rubber–filler–aggregate ternary system. Utilizing surface free energy theory, we quantitatively analyze the surface energy components, adhesion work, and debonding energy of mineral fillers in rubberized asphalt systems. Furthermore, molecular dynamics simulations are employed to elucidate the influence of both mineral filler and aggregate types on interfacial adhesion properties, ultimately revealing the fundamental bonding mechanisms at the rubberized mastic–aggregate interface. These findings provide novel insights into the formation of interfacial bond strength, with the complete methodological framework illustrated in Figure 1.

## 2. Materials and Methods

### 2.1. Materials

#### 2.1.1. Asphalt Binder

This study utilizes Panjin 90# (penetration grade) as the base material, with its physical properties listed in Table 1.

#### 2.1.2. Rubber Powder

The physical indices of rubber powder are shown in Table 2.

#### 2.1.3. Mineral Powder

In this work, the mineral powder used is limestone mineral powder, and the relevant test indices are shown in Table 3.

**Figure 2:**
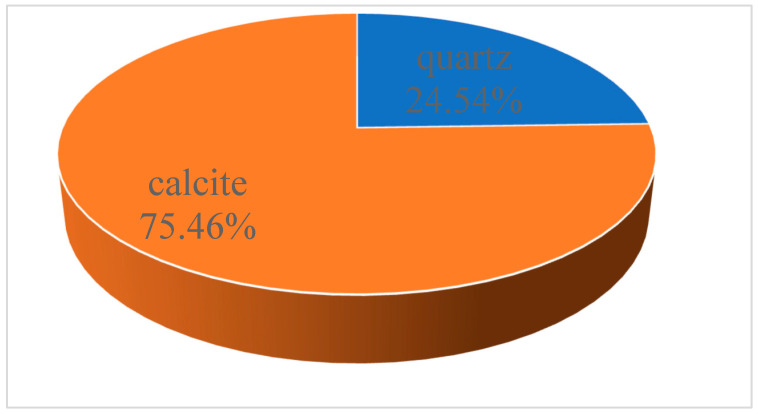
XRD test results for limestone.

The chemical composition and mineralogical composition of the limestone were analyzed using X-ray diffraction (XRD) and X-ray fluorescence spectrometry (XRF), and the results are shown in Figure 2 and Table 4.

From Table 4, it was found that the CaO content and SiO_2_ content of the limestone used were 52.63% and 26.56%, respectively. From Figure 2, it was found that quartzite and calcite are the main mineral components of limestone. To minimize the influence of other components, calcite (CaCO_3_) and quartz (SiO_2_) were used as the subjects of this work.

### 2.2. Preparation of Rubber Asphalt (Mastic)

**Figure 3:**
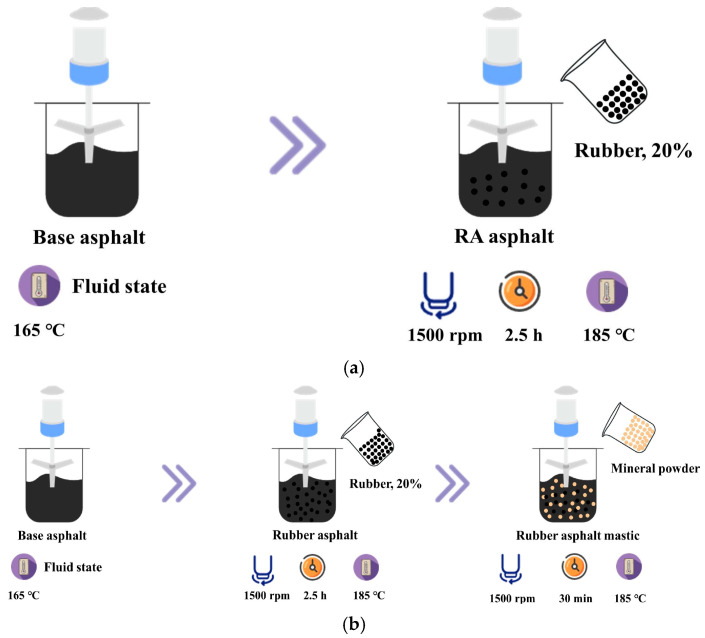
Preparation process of rubber asphalt (mastic) ((**a**): rubber asphalt preparation process; (**b**): rubber asphalt mastic preparation process).

The preparation process of rubber asphalt and rubber asphalt mastic: ① After the base asphalt was heated to a fluid state at 165 °C, 20% (weight of base asphalt) of the rubber powder was weighed and added to the base asphalt. Stir the asphalt at 185 °C and 1500 rpm for 2.5 h to obtain rubber asphalt. ② A certain amount of mineral powder was weighed and added to the rubber asphalt and was stirred at 1500 r/min for 30 min to obtain different powder-to-gum ratios (0.6, 0.8, and 1.0) of rubber asphalt mastic. The specific preparation process is shown in Figure 3.

### 2.3. Surface Free Energy Theory

According to reference [21], Equation (1) mathematically describes the interaction between the polar and dispersive constituents of a material’s surface energy.

(1) $$\gamma =\gamma^{d}+\gamma^{p}=\gamma^{d}+2\sqrt{\gamma^{+}\gamma^{-}}$$

where *γ^p^* is the polar component, *γ^d^* is the dispersive component, *γ^+^* is the Lewis acid, and *γ^−^* is the Lewis alkali.

Young’s equation (Equation (2)) [22] describes the correlation between the surface energy of asphalt–aggregate systems and their contact angle (θ).

(2) $$\gamma_{a}\cos\theta =\gamma_{s}-\gamma_{as}$$

where *γ_a_* is the surface energy of the asphalt binder, *γ_s_* is the surface energy of the aggregate, and *γ_as_* is the surface energy of the asphalt mixture.

The interfacial adhesion performance of asphalt and aggregate can be evaluated by the adhesion work (*W*_as_), calculated using Equation (3) [23].

(3) $$W_{as}=\gamma_{a}(1+\cos\theta )=2\sqrt{\gamma_{a}^{d}\gamma_{s}^{d}}+2\sqrt{\gamma_{a}^{+}\gamma_{s}^{-}}+2\sqrt{\gamma_{a}^{-}\gamma_{s}^{+}}$$

The adhesion work (W_as_) represents asphalt–aggregate bonding in dry conditions, whereas wet conditions typically weaken this interface. The peeling work, defined in Equation (4) [24], quantifies the adhesion under moisture exposure.

(4) $$\begin{array}{ll} W_{asw} & =2\sqrt{\gamma_{a}^{d}\gamma_{w}^{d}}+2\sqrt{\gamma_{s}^{d}\gamma_{w}^{d}}+2\sqrt{\gamma_{w}^{+}}\left(\sqrt{\gamma_{a}^{-}}+\sqrt{\gamma_{s}^{-}}\right)+2\sqrt{\gamma_{w}^{-}}\left(\sqrt{\gamma_{a}^{+}}+\sqrt{\gamma_{s}^{+}}\right)\\ & -2\gamma_{w}^{d}-2\sqrt{\gamma_{a}^{d}\gamma_{s}^{d}}-2\sqrt{\gamma_{a}^{+}\gamma_{s}^{-}}-2\sqrt{\gamma_{a}^{-}\gamma_{s}^{+}}-4\sqrt{\gamma_{w}^{-}\gamma_{w}^{+}} \end{array}$$

To calculate the surface free energy of asphalt, three solvents: distilled water, glycerin, and formamide are used as known solvents in this paper. The surface energy parameters of the three solvents are shown in Table 5.

The surface energy parameters and components of the aggregate are shown in Table 6.

## 3. Molecular Dynamics Simulation

### 3.1. Construction of the Rubber Asphalt Mastic Molecular Model

#### 3.1.1. Molecular Model of Asphalt

**Figure 4:**
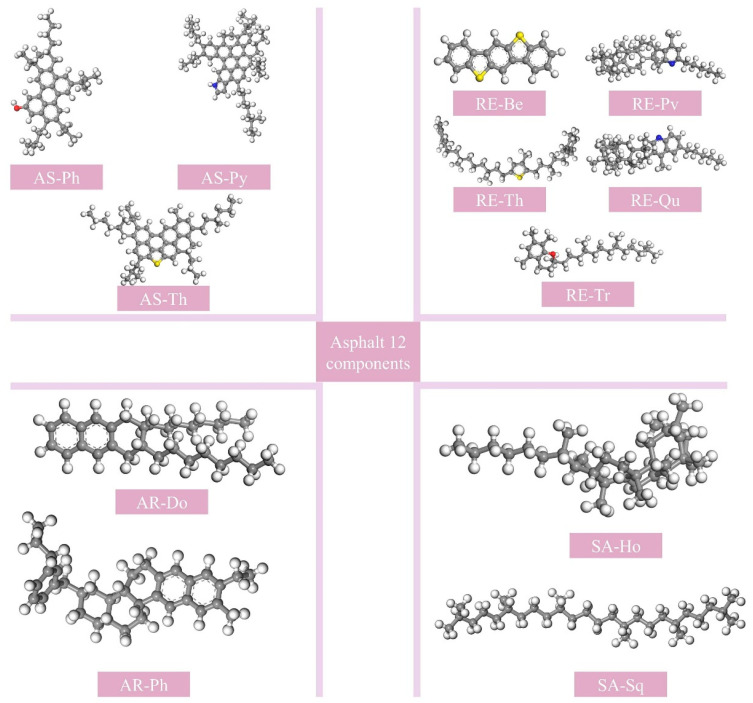
The 12-component molecular model of asphalt.

The AAA-1 model proposed by Li and Greenfield has been widely used to represent an asphalt binder [11,25], and its rationality and reliability have long been proven [26,27,28]. Therefore, this paper selects the AAA-1 model to represent the molecular model of the asphalt binder. The 12-component molecular model is shown in Figure 4, and the number of molecules is shown in Table 7.

#### 3.1.2. Molecular Model of Asphalt

**Figure 5:**
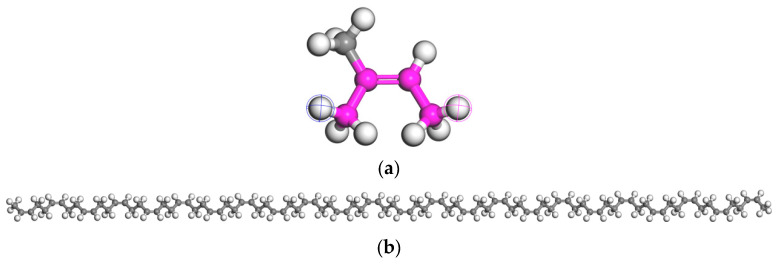
Molecular model of rubber ((**a**): isoprene monomer molecule; (**b**): NR molecule).

In general, rubber tires are mainly composed of natural rubber (NR), butadiene rubber (BR), and styrene-butadiene rubber (SBR), with the largest percentage of natural rubber content [11,29,30]. Therefore, NR is used in this paper to represent the rubber molecule. NR is formed by the polymerization of an isoprene monomer with a polymerization degree of 24. Figure 5 illustrates the isoprene monomer molecular model and the NR molecular model.

#### 3.1.3. Molecular Model of Mineral Powder

Mineral powder is an important component of asphalt mastic. In this paper, the SiO_2_ cluster structure and CaCO_3_ cluster structure are used to represent the most common acidic mineral powders (granite) and alkaline mineral powders (limestone). The crystal parameters of SiO_2_ and CaCO_3_ are shown in Table 8. 

**Figure 6:**
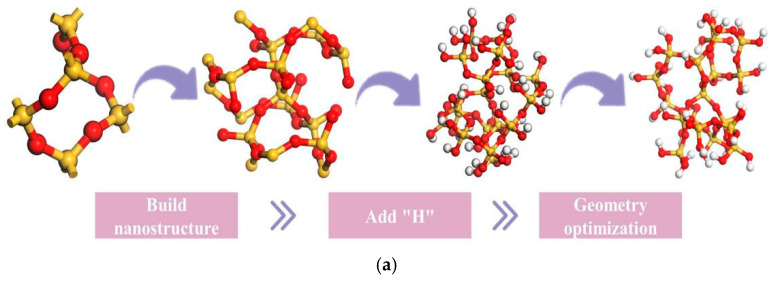

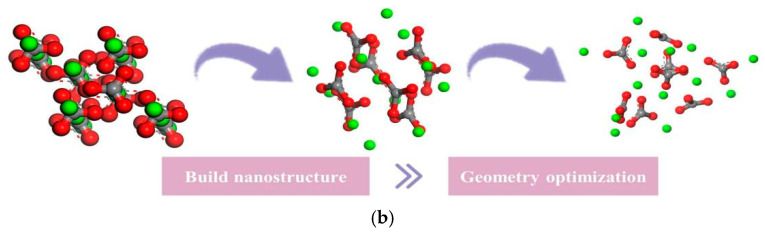
The building process of the mineral powder molecular model ((**a**): SiO_2_ cluster structure; (**b**): CaCO_3_ cluster structure).

The SiO_2_ and CaCO_3_ cluster structures were constructed using MS based on the SiO_2_ and CaCO_3_ crystal models in Table 8. The radius of the cluster structure constructed in this paper is 5 Å. Figure 6 shows the construction process of the SiO_2_ and CaCO_3_ cluster structures.

#### 3.1.4. Molecular Model of Rubber Asphalt Mastic

The molecular model of the rubber-modified asphalt mastic system was constructed using the amorphous cell module of 2021 MS software. The number of NR molecules, SiO_2_ cluster structure molecules, and CaCO_3_ cluster structure molecules for the molecular model of rubber asphalt mastic are shown in Table 9.

**Figure 7:**
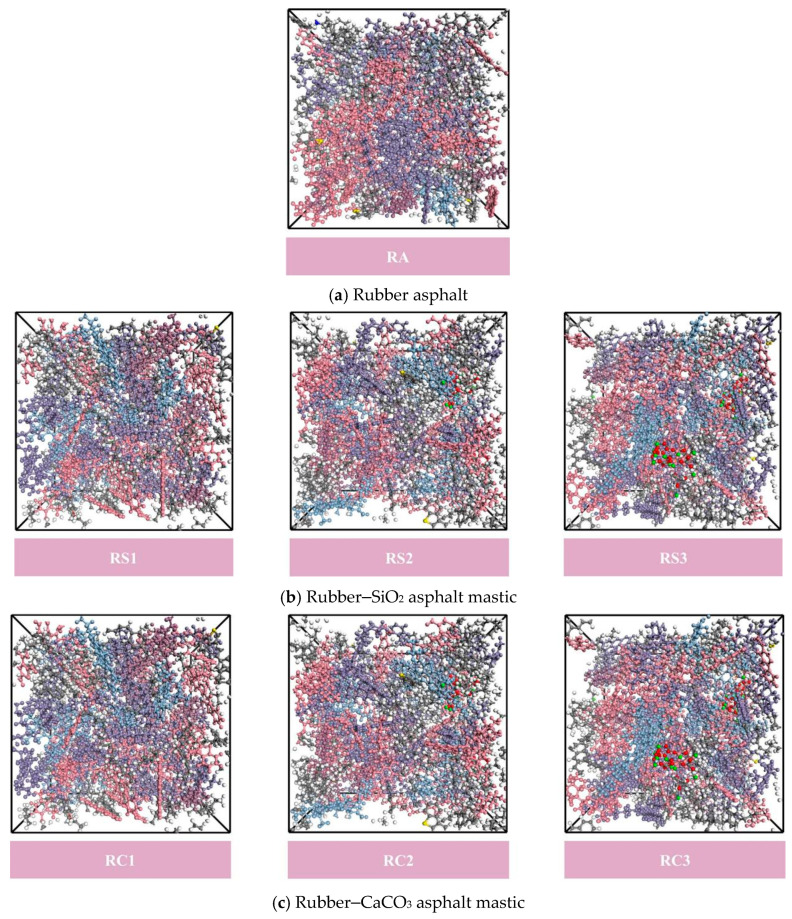
Molecular model of rubber asphalt mastic.

The rubber asphalt mastic was constructed based on the number of molecules from Table 1 and Table 3, as shown in Figure 7.

### 3.2. Construction of the Rubber Asphalt Mastic-Aggregate Molecular Model

**Figure 8:**
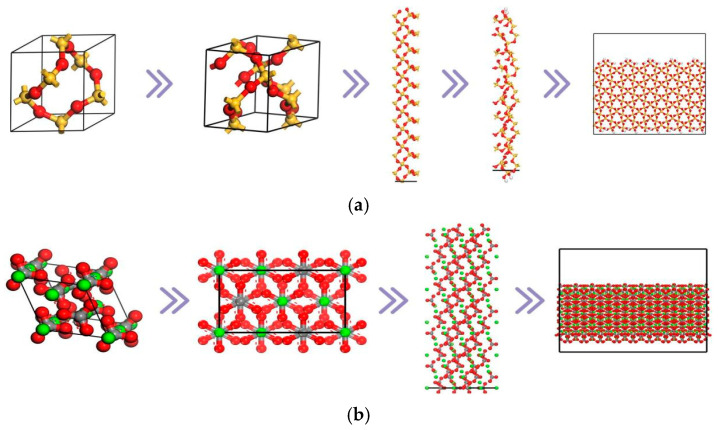
Construction process of the aggregate layer ((**a**) SiO_2_ aggregate layer; (**b**) CaCO_3_ aggregate layer).

The main components of aggregate for road construction contain quartz (SiO_2_) and calcite (CaCO_3_); thus, SiO_2_ and CaCO_3_ are used to represent acidic and alkaline aggregates, respectively. According to the parameters of the SiO_2_ and CaCO_3_ crystals in Table 8, the SiO_2_ and CaCO_3_ 3D models were established. Secondly, the SiO_2_ and CaCO_3_ crystals were cut along the [1, 0, 0] direction. Finally, to make the aggregate layer bonded better with the asphalt mastic layer, the SiO_2_ and CaCO_3_ 3D crystal models were orthogonalized. Figure 8 shows the construction process of the aggregate layer.

**Figure 9:**
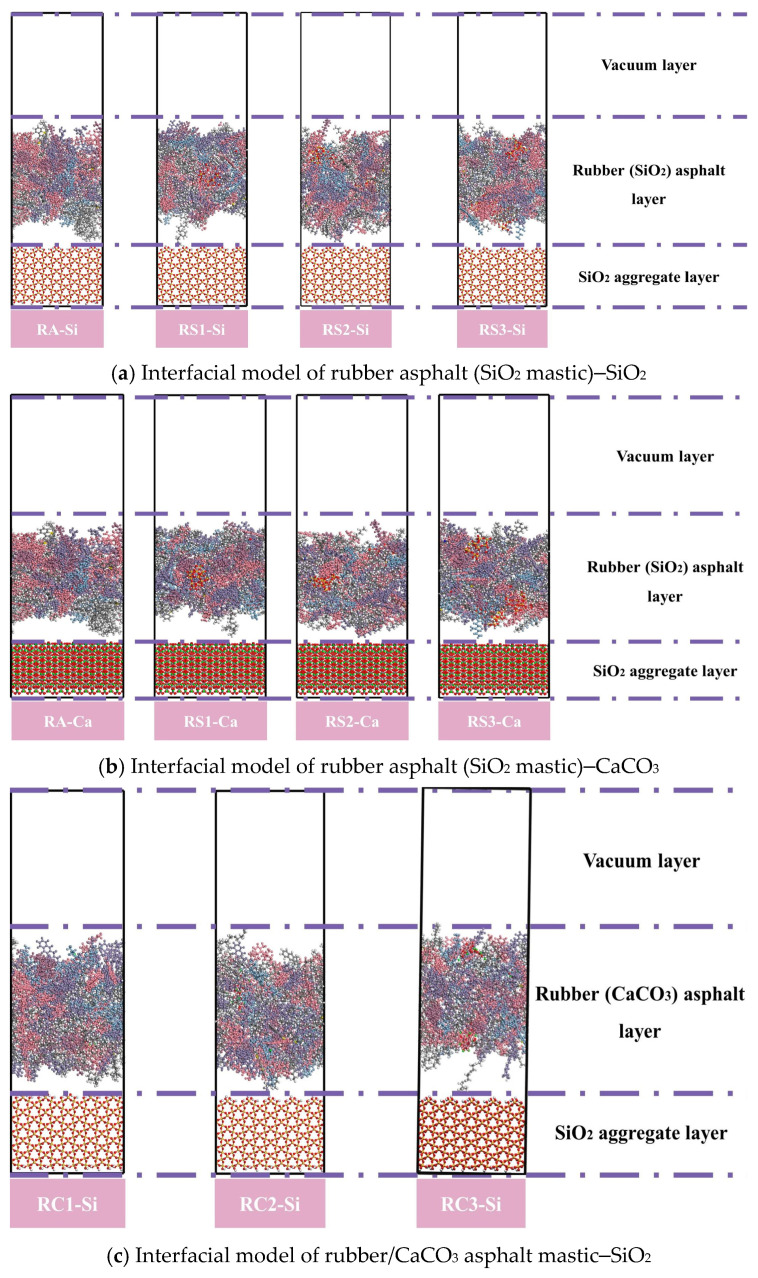

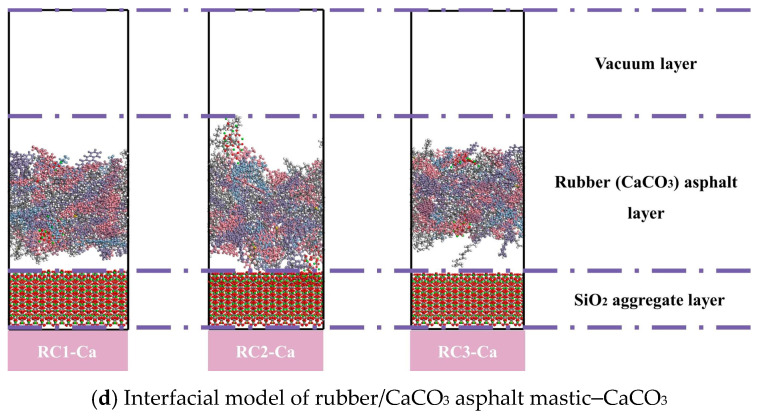
Interface model of rubber asphalt mastic–aggregate.

The rubber asphalt mastic–aggregate interface model is obtained by adding the rubber asphalt mastic above the aggregate layer. A vacuum layer of 50 Å is added above the rubber asphalt mastic to avoid cyclicity effects. The 3D interface model of rubber asphalt mastic–aggregate is illustrated in Figure 9.

### 3.3. Rubber Asphalt Mastic–Water–Aggregate Interface Molecular Model

**Figure 10:**
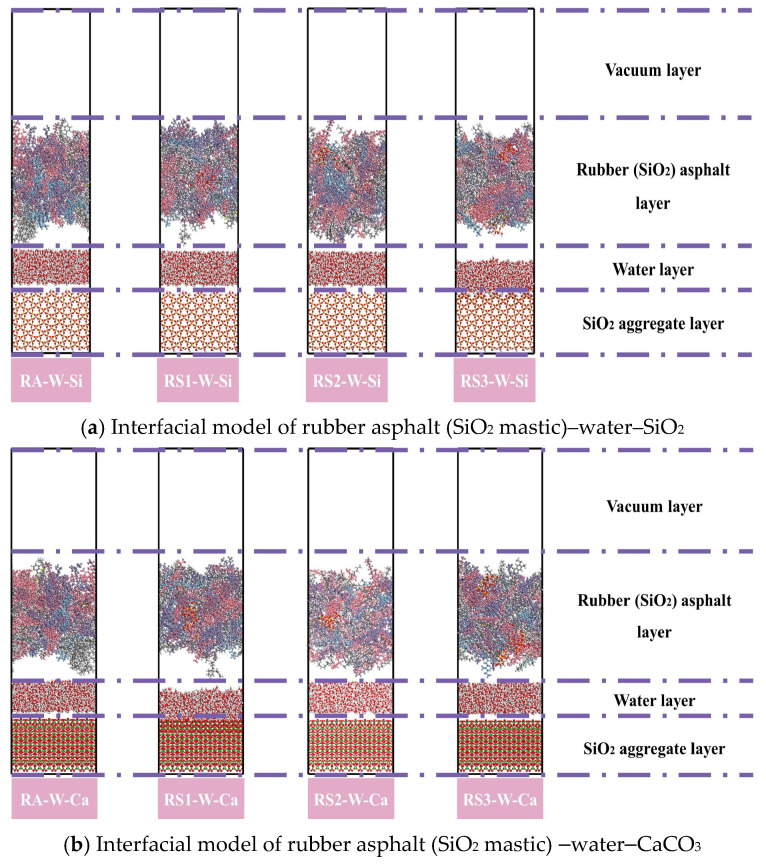

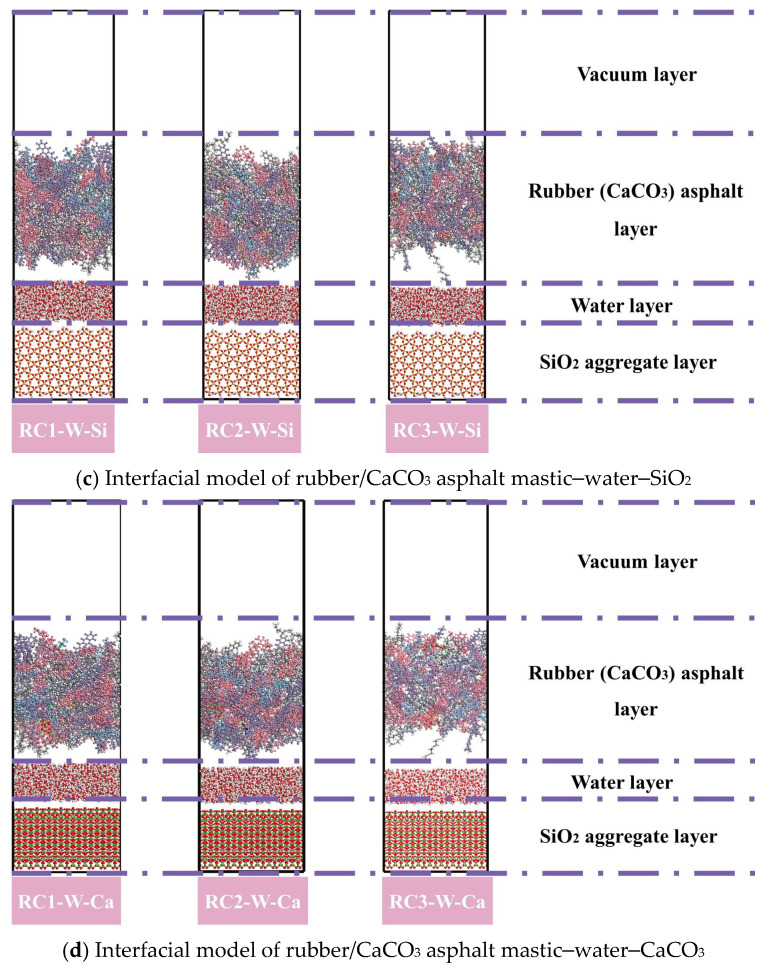
Interface model of rubber asphalt mastic–water–aggregate.

This study constructed a three-layer interface model of rubber asphalt mastic–water–aggregate to examine the debonding behavior between rubber asphalt mastic and aggregate in the presence of water, as shown in Figure 10.

### 3.4. Simulation Methods

This paper used Materials Studio (version 2021) throughout to perform molecular dynamics calculations. This study accomplished the molecular dynamics simulation process under 500 ps NVT conditions. It should be noted that the selected force field in this paper is the COMPASS II force field [18,31,32], and the whole simulation is carried out at 298 K. It should be noted that, in order to ensure the accuracy of the results, the simulation is repeated three times for all models in this paper, and the results are averaged.

### 3.5. Evaluation Indicators

#### 3.5.1. Adhesive Work

The adhesion work represents the energy required to separate the asphalt from the aggregate surface, which is calculated as shown in Equation (5) [33].

(5) $$W_{\mathrm{adhesion}}=-\frac{E_{\mathrm{int}}}{A}=-\frac{E_{as}+E_{ag}-E_{as+ag}}{A}$$

where *W*_adhesion_ is the adhesion work, *E*_int_ is the interaction energy between asphalt and aggregate, *E*_as_ is the potential energy of asphalt, *E*_ag_ is the potential energy of aggregate, *E*_as+ag_ is the potential energy of the interface between asphalt and aggregate, and *A* is the area of contact between asphalt and aggregate interface.

#### 3.5.2. Debonding Work

The presence of water at the asphalt–aggregate interface makes it easier for the asphalt to separate from the aggregate interface. This paper uses debonding work to evaluate the bonding characteristics between asphalt and aggregate in the presence of water, as shown in Equation (6) [34].

(6) $$W_{\mathrm{debonding}}=-\frac{E_{as-wa}+E_{ag-wa}-E_{as-ag}}{A}$$

where W_debonding_ is the debonding work, *E*_as–wa_ is the interaction energy between asphalt and water, *E*_ag–wa_ is the interaction energy between aggregate and water, and *E*_as–ag_ is the interaction energy between asphalt and aggregate.

#### 3.5.3. Energy Ratio (ER)

It has been shown that the energy ratio (ER) can represent the ability of the asphalt–aggregate interfacial system to resist moisture damage, as shown in Equation (7) [35].

(7) $$ER=\left|\frac{W_{\mathrm{adhesion}}}{W_{\mathrm{debonding}}}\right|$$

## 4. Results and Discussion

### 4.1. Results Analysis of Surface Free Energy

#### 4.1.1. Surface Energy Component

**Figure 11:**
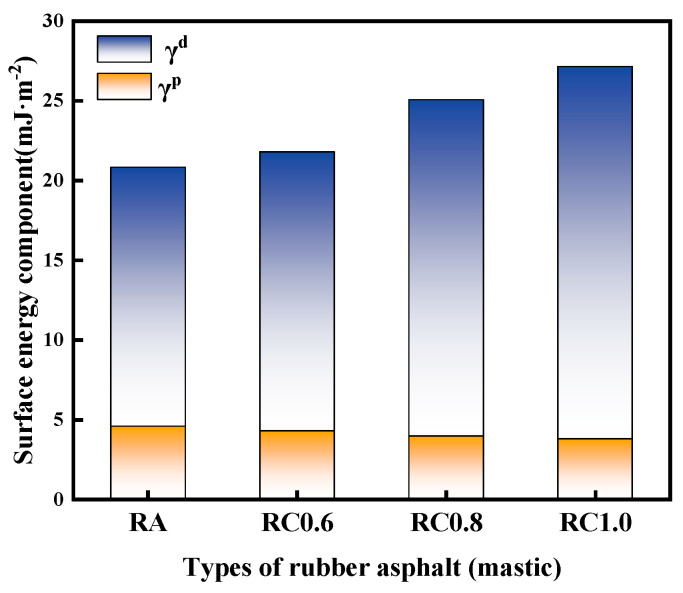
Calculated results of the surface energy and its components (Note: RC0.6, RC0.8, and RC1.0 represent rubber asphalt mastic with a powder/asphalt ratio of 0.6, 0.8, and 1.0, respectively).

Figure 11 demonstrates the surface energy of rubber asphalt (mastic) and its components. In Figure 11, the dispersive component of rubber asphalt increases gradually with the increase in mineral powder content. The dispersive components of RA, RC0.6, RC0.8, and RC1.0 are 16.25 mJ·m^−2^, 17.51 mJ·m^−2^, 21.11 mJ·m^−2^, and 23.37 mJ·m^−2^, respectively. In contrast, the polar component of rubber asphalt decreases with the increasing mineral powder content. The polar components of RA, RC0.6, RC0.8, and RC1.0 are 4.58 mJ·m^−2^, 4.29 mJ·m^−2^, 3.97 mJ·m^−2^, and 3.79 mJ·m^−2^, respectively. The experimental results demonstrate that the limestone mineral filler significantly enhances the surface energy of rubber-modified asphalt. This improvement stems from the calcite (CaCO_3_) composition of limestone, where the charged CaCO_3_ surface facilitates asphalt molecule adsorption through electrostatic interactions. This interfacial phenomenon substantially increases the dispersive component of the rubberized asphalt system [36].

#### 4.1.2. Adhesion Work

**Figure 12:**
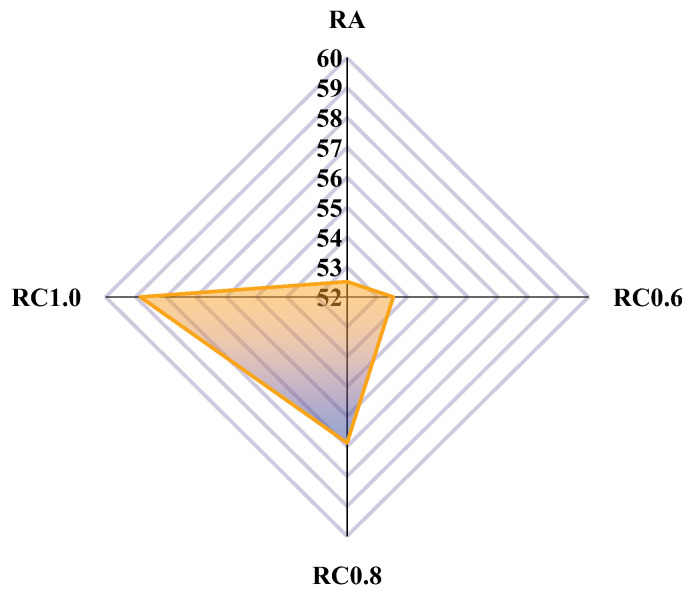
Calculated results of the adhesion work.

Adhesion work is an indication of the adhesion properties of the asphalt and aggregate system. The greater the work of adhesion, the better the adhesion between the asphalt and aggregate. The adhesion work for RA, RC0.6, RC0.8, and RC1.0 was calculated according to Equation (3), and the results are shown in Figure 12.

Figure 12 shows the adhesion work for RA, RC0.6, RC0.8, and RC1.0, respectively. The adhesion work of RA, RC0.6, RC0.8, and RC1.0 were 52.51 mJ·m^−2^, 53.48 mJ·m^−2^, 56.88 mJ·m^−2^, and 58.85 mJ·m^−2^, respectively. This indicates that mineral powder can effectively improve the adhesion work of rubber asphalt. This further confirms Wang and Pasandín’s conjecture [37,38]. After the rubber asphalt mastic is added to the mineral powder, the active sites on the surface of the mineral powder particles come into contact with the asphalt molecules. These sites provide a large number of binding sites, which enhance the physical and chemical bonding between asphalt and mineral powder, thus improving the adhesion work between asphalt and aggregate.

#### 4.1.3. Peeling Work

**Figure 13:**
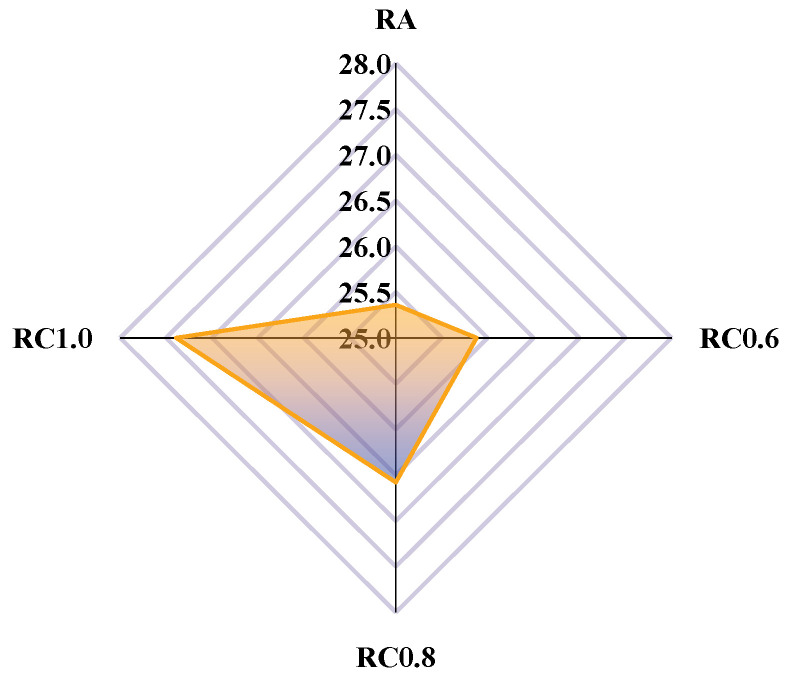
Calculated results of the peeling work.

The peeling work represents the work required to remove the asphalt from the aggregate surface in the presence of water. The greater the peeling work, the more easily the water displaces the asphalt film and the poorer the adhesion of the asphalt and aggregate. The peeling work for RA, RC0.6, RC0.8, and RC1.0 was calculated according to Equation (4), and the results are shown in Figure 13.

### 4.2. Interaction Energy Between Rubber Asphalt (Mastic)–Aggregate

**Figure 14:**
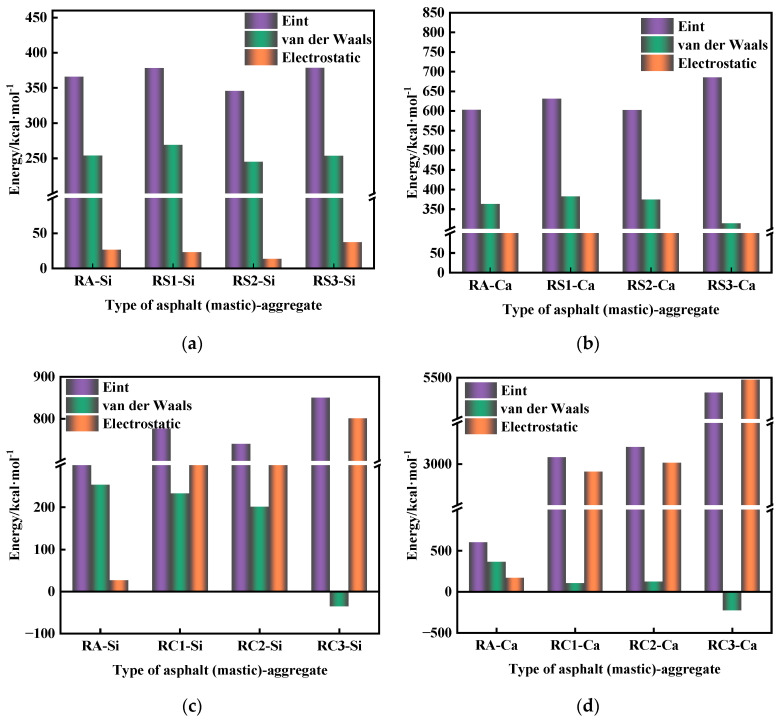
Interaction energy, van der Waals forces, and electrostatic energy of rubber asphalt (mastic)–aggregate. (**a**) Model of the rubber (SiO_2_ mineral powder)–SiO_2_ aggregate interface, (**b**) model of the rubber (SiO_2_ mineral powder)–CaCO_3_ aggregate interface, (**c**) model of the rubber (CaCO_3_ mineral powder)–SiO_2_ aggregate interface, and (**d**) model of the rubber (CaCO_3_ mineral powder)–CaCO_3_ aggregate interface.

The interaction energy consists of chemical bonding energy and non-bonded forces. The non-bonding energy consists of van der Waals forces and electrostatic interactions [39,40]. Figure 14 illustrates the asphalt–aggregate interface’s interaction energy, van der Waals forces, and electrostatic energy.

**Figure 15:**
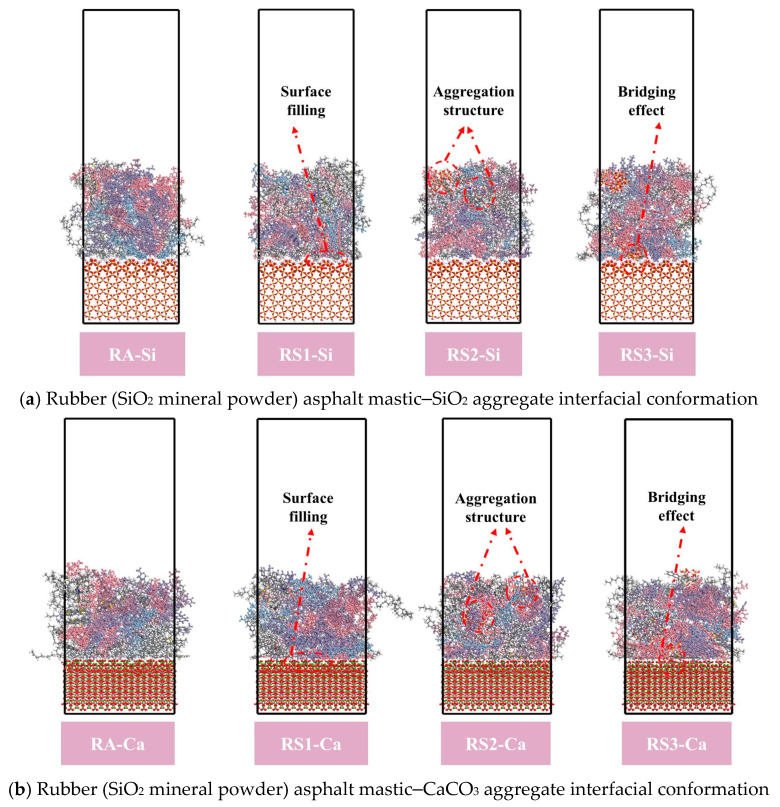
Rubber asphalt mastic–aggregate interfacial conformation.

Figure 14a,b show the interaction energy, van der Waals force, and electrostatic energy of rubber asphalt (SiO_2_ mineral powder)–SiO_2_ aggregate and rubber asphalt (SiO_2_ mineral powder)–CaCO_3_ aggregate, respectively. The interaction energy of RA–Si, RS1–Si, RS2–Si, and RS3–Si was 365.929 kcal/mol, 378.169 kcal/mol, 345.5 kcal/mol, and 378.522 kcal/mol, respectively. The interaction energy of RA–Ca, RS1–Ca, RS2–Ca, and RS3–Ca was 602.756 kcal/mol, 630.970 kcal/mol, 602.096 kcal/mol, and 685.446 kcal/mol, respectively. The above results show that the low concentration of the SiO_2_ cluster structure can improve the interaction energy between rubber asphalt and aggregate. This may be that a small amount of SiO_2_ cluster structure not only increases the surface activity of asphalt but also increases the contact area between rubber asphalt and aggregate, which makes the asphalt and aggregate combine more closely, fills up the tiny pores and unevenness on the surface of the aggregate, and thus increases the interaction energy between rubber asphalt and aggregate. As the number of SiO_2_ cluster structures increases, the interaction energy between rubber asphalt mastic and aggregate is decreased, as shown in Figure 14a,b. As the concentration of SiO_2_ cluster increases, the “H” on the surface of these SiO_2_ clusters forms hydrogen bonding with asphaltene and resin molecules to form a larger aggregation structure, which impedes the contact between the rubber asphalt and aggregate and reduces the interaction energy between the asphalt mastic and the aggregate. In addition, the high concentration of the SiO_2_ cluster structure rather increases the interaction energy between rubber asphalt and aggregate. This is mainly due to the SiO_2_ cluster structure forming a bridging effect between the rubber asphalt and aggregate, increasing the van der Waals force between the asphalt mastic and aggregate [41], and then improving the interaction between the asphalt mastic and the aggregate, as shown in Figure 15. These results indicate that the interaction energy between asphalt mastic and aggregate depends on the type of aggregate [42].

Figure 14c,d show the interaction energy, van der Waals force, and electrostatic energy of rubber asphalt (CaCO_3_ mineral powder)–SiO_2_ aggregate and rubber asphalt (CaCO_3_ mineral powder)–CaCO_3_ aggregate, respectively. The interaction energy of RA–Si, RC1–Si, RC2–Si, and RC3–Si was 365.929 kcal/mol, 776.501 kcal/mol, 740.282 kcal/mol, and 850.202 kcal/mol, respectively. The interaction energy of RA–Ca, RC1–Ca, RC2–Ca, and RC3–Ca was 602.756 kcal/mol, 3083.881 kcal/mol, 3209.635 kcal/mol, and 5319.691 kcal/mol, respectively. For example, compared to RS1–Si and RS1–Ca, RC1–Si and RC1–Ca increased by 105% and 389%, respectively. This is mainly attributed to the presence of carbonate ions in the structure of CaCO_3_ clusters, which generate electrostatic interactions when they come into contact with asphaltene molecules and resin molecules, enhancing the interaction between the rubberized asphalt and the aggregate.

**Figure 16:**
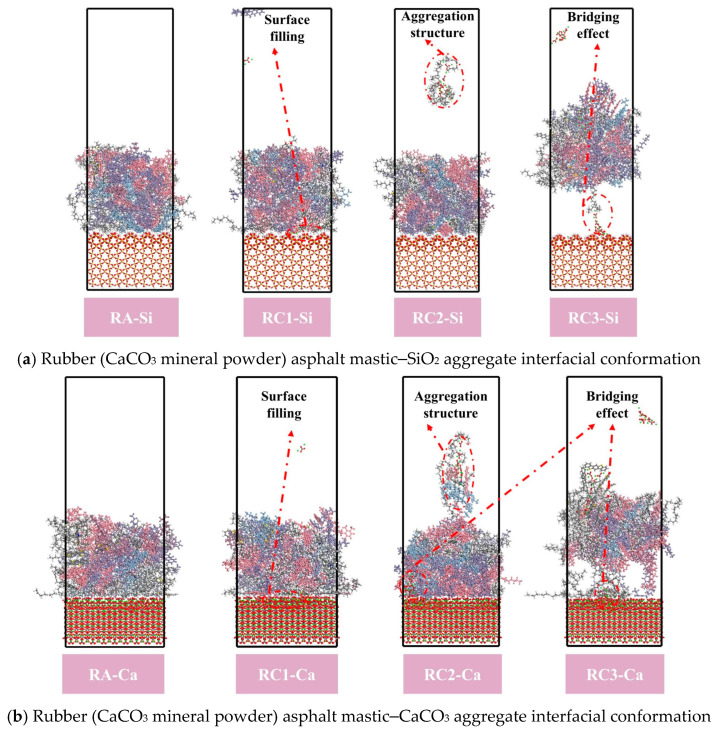
Rubber asphalt mastic–aggregate interfacial conformation.

As for the SiO_2_ structure, which is mainly composed of Si-O bonds, the charge distribution is relatively uniform, and its interaction with asphaltene molecules and resin molecules is primarily van der Waals forces, so the interaction between the asphalt mastic and aggregate can be dominated by van der Waals forces, as shown in Figure 14. The van der Waals force between asphalt mastic and aggregate is negative under a high concentration of CaCO_3_ filler. The van der Waals force of RC3–Si and RC3–Ca are −35.345 kcal/mol and -228.519 kcal/mol, respectively. This phenomenon may be due to the high concentration of CaCO_3_ and asphalt molecules to form a dense network structure, and CaCO_3_ acts as a bridge to shorten the contact distance between the asphalt mortar molecules and the aggregate, as shown in Figure 16. In addition, CaCO_3_ shows high polarity, resulting in the interaction force between asphalt mastic and aggregate being dominated by electrostatic energy. At this time, the van der Waals force exhibits a repulsive effect, resulting in a negative van der Waals force.

### 4.3. Adhesion Behavior Between Asphalt and Aggregate Under Dry Conditions

**Figure 17:**
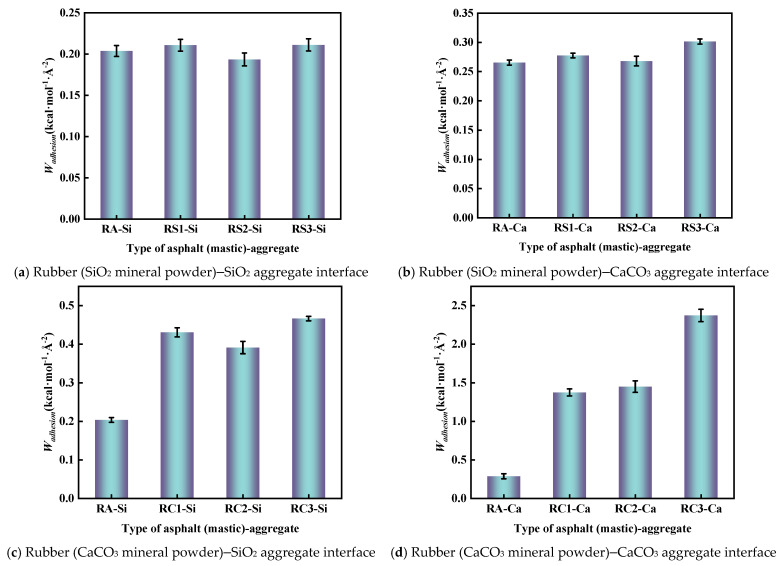
Adhesion work of the rubber asphalt (mastic)–aggregate.

To evaluate the energy required for the separation of rubber asphalt mastic from the aggregate surface under the dry conditions, the adhesion work of rubber asphalt (mastic) was calculated according to Equation (5). The results of the calculation are shown in Figure 17.

As can be seen from Figure 17, the adhesion work between rubber asphalt mastic and aggregate was significantly improved by the addition of mineral powder. For example, the adhesion work of RA–Si, RS1–Si, and RC1–Si was 0.20 kcal·mol^−1^·Å^−2^, 0.21 kcal·mol^−1^·Å^−2^, and 0.42 kcal·mol^−1^·Å^−2^, respectively. The adhesion work of RA–Ca, RS1–Ca, and RC1–Ca was 0.26 kcal·mol^−1^·Å^−2^, 0.27 kcal·mol^−1^·Å^−2^, and 1.34 kcal·mol^−1^·Å^−2^, respectively. However, the improvement effect of SiO_2_ filler on the adhesion work between rubber asphalt and aggregate is much lower than that of CaCO_3_ filler. This is because the surface of CaCO_3_ filler has a positive and negative charge, which can produce a strong electrostatic attraction, significantly enhancing the interfacial bonding strength between asphalt mastic and aggregate. At the same time, the high surface energy of the CaCO_3_ surface makes it form stronger adhesion with asphalt, which further enhances the adhesion work between rubber asphalt mastic and the aggregate system. In contrast, SiO_2_ filler mainly relies on weak van der Waals forces, and its surface chemical inertness makes it less effective in enhancing the adhesion between the rubber asphalt mastic and the aggregate. Therefore, although SiO_2_ filler can also enhance the adhesion work, its effect is inferior to that of CaCO_3_ filler. Consistent with previous studies, the adhesion work of rubberized asphalt mastic with CaCO_3_ aggregate is much higher than that of rubber asphalt mastic with SiO_2_ aggregate [23,43].

### 4.4. Debonding Work Between Asphalt and Aggregate Under Wet Conditions

**Figure 18:**
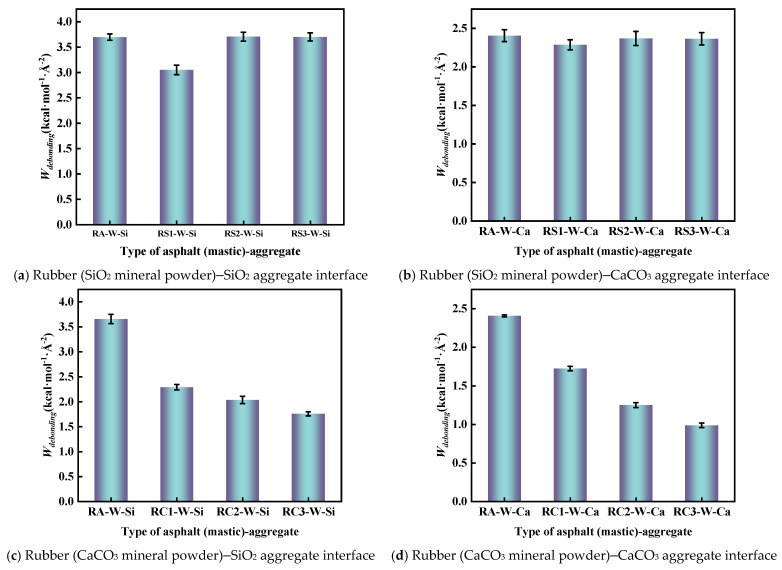
Debonding work of the rubber asphalt (mastic)–aggregate.

To evaluate the debonding behavior of asphalt from the aggregate surface in the presence of moisture, the debonding work of the rubber asphalt (mastic)–water–aggregate system was calculated using Equation (6), the results are shown in Figure 18. The greater the debonding work, the easier it is to be destroyed by moisture.

**Figure 19:**
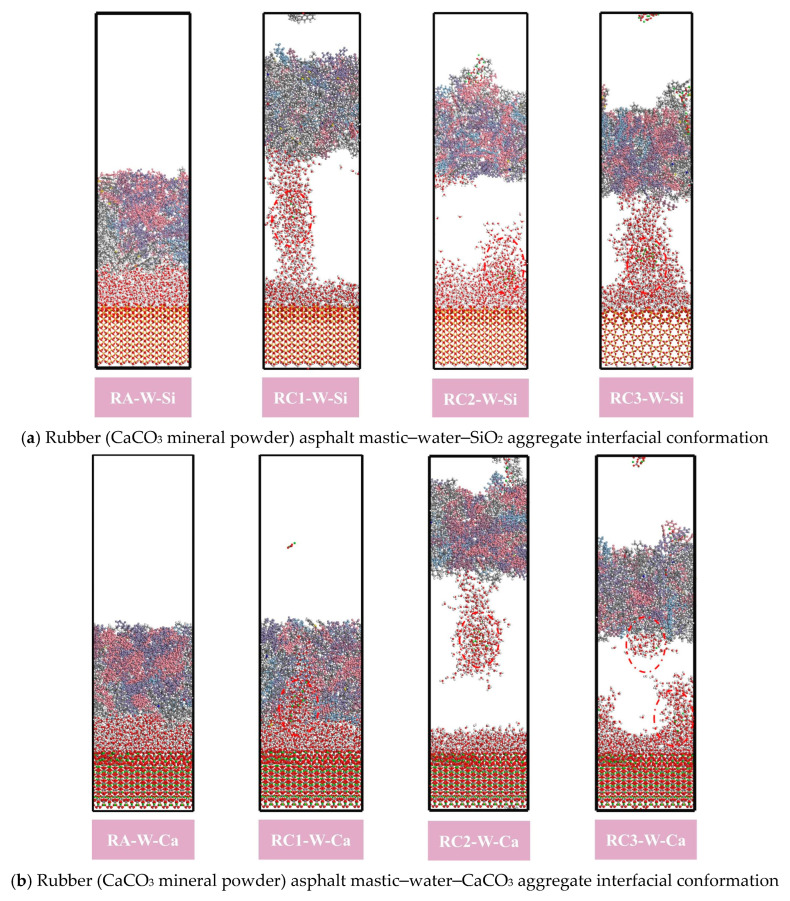
Rubber asphalt mastic–water–aggregate interfacial conformation.

From Figure 18, it is found that the addition of SiO_2_ and CaCO_3_ fillers can effectively reduce the debonding work between rubber asphalt–aggregate. For example, the debonding work of RA–W–Si, RS1–W–Si, and RC1–W–Si was 3.66 kcal·mol^−1^·Å^−2^, 3.12 kcal·mol^−1^·Å^−2^, and 2.29 kcal·mol^−1^·Å^−2^, respectively. This indicates that SiO_2_ and CaCO_3_ fillers can improve the moisture damage resistance of rubber asphalt. However, SiO_2_ fillers are very limited in improving the ability of rubberized asphalt to resist moisture damage. This is because the surface of CaCO_3_ particles has positive and negative charges. The electronegative atoms (N, S, and O) present in both the crumb rubber and asphalt binder generate substantial electrostatic attraction forces. This molecular-level interaction enables rubber-modified asphalt to maintain strong interfacial bonding with aggregates even under moisture-exposed conditions. Furthermore, CaCO_3_ particles exhibit high surface energy and pronounced hydrophilicity, enabling them to establish robust interfacial interactions with both asphalt and water molecules. A portion of water molecules become adsorbed onto the CaCO_3_ particle surfaces, forming a structured water film that effectively inhibits further water molecule diffusion (Figure 19). These synergistic effects collectively enhance the interfacial stability, thereby significantly improving the moisture damage resistance of rubber-modified asphalt systems.

**Figure 20:**
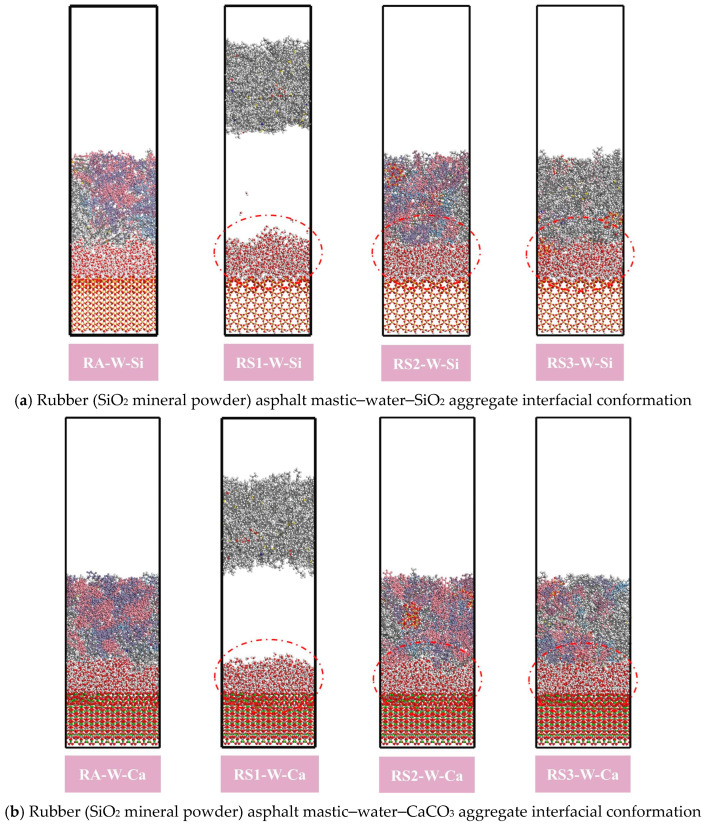
Rubber asphalt mastic–water–aggregate interfacial conformation.

However, for SiO_2_ particles, the SiO_2_ surface relies on van der Waals forces to interact with asphalt and water molecules, and these forces are easily interfered with and weakened by water molecules. In addition, due to the lack of electrostatic forces, SiO_2_ is unable to provide sufficiently strong interfacial bonding in the presence of water, making it easier for water molecules to penetrate into the interface, thus reducing the resistance to moisture damage. This is attributed to the fact that the weak electrical properties of SiO_2_ cause water molecules to collect between the rubber asphalt mastic and the aggregate, forming a thick water film, as shown in Figure 20. This water film forms an isolation layer between rubber asphalt mastic and aggregate, weakening the bond between rubber asphalt and aggregate.

### 4.5. Energy Ratio Between Asphalt and Aggregate

**Figure 21:**
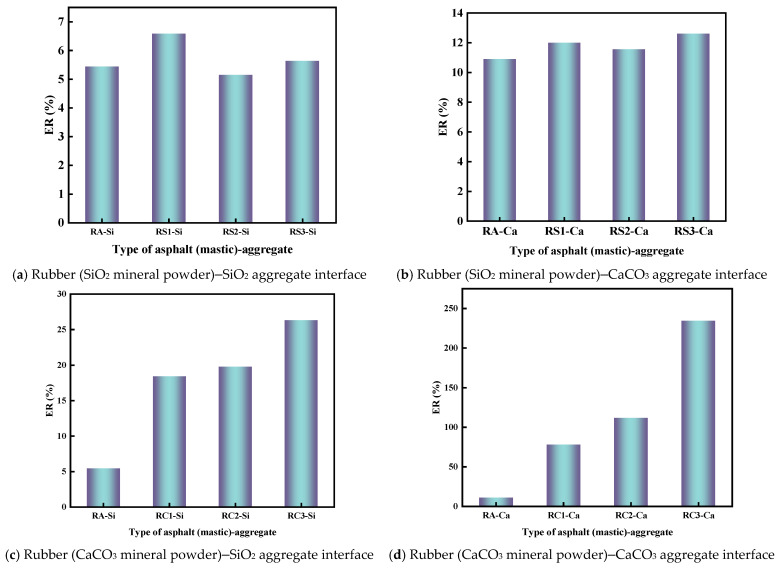
ER of rubber asphalt (mastic)–aggregate.

The energy ratio (ER) is a comprehensive parameter that characterizes the bonding and debonding behavior between rubberized asphalt mastic and aggregate, and the ER values of rubberized asphalt mastic and aggregate were calculated using Equation (7), and the results are shown in Figure 21.

In Figure 21, the ER values of rubber asphalt mastic–SiO_2_ aggregate were lower than those of rubber asphalt mastic–CaCO_3_ aggregate. A similar result can be found in previous studies [43]. For example, the ER values for RS1–Si and RS1–Ca are 6.59% and 12.01%. The type of mineral powder greatly affects the ER value of rubber asphalt mastic–aggregate, as shown in Figure 21. The ER values for RS1–Si and RC1–Si were 6.59% and 18.42%, respectively. The ER values for RS1–Ca and RC1–Ca were 12.01% and 77.85%, respectively. This indicates that the debonding of rubberized asphalt–aggregate by CaCO_3_ mineral powder is much better than the debonding of rubber asphalt–aggregate by SiO_2_ mineral powder. In addition, the rubber asphalt mastic–CaCO_3_ aggregate has better anti-debonding properties with the synergistic effect of CaCO_3_ mineral powder and CaCO_3_ aggregate. This property is one of the factors why limestone is often used as a road building material.

## 5. Conclusions

This work investigated the adhesion properties and debonding behavior between rubber asphalt (mastic) and aggregate using surface free energy theory and molecular dynamics. Some key conclusions were drawn:

(1) Limestone mineral powder enhances the surface energy and dispersive component of asphalt and reduces the polar component of asphalt. It improves the adhesion work between rubber asphalt and aggregate and inhibits water molecules from displacing the asphalt film between rubber asphalt and aggregate.

(2) The type of mineral powder has a significant effect on the interaction energy between rubber asphalt–aggregate. The interaction energy between rubber asphalt/SiO_2_–aggregate is dominated by van der Waals forces, and the interaction energy between rubber asphalt/CaCO_3_–aggregate is dominated by electrostatic forces.

(3) Mineral powder plays three important roles between rubber asphalt mastic and aggregate, and they are to (a) promote rubber asphalt mastic to fill the void on the surface of aggregate, (b) form a cluster structure with asphalt molecules in asphalt to increase the direct contact area between rubber asphalt and aggregate, and (c) act as a bridge between rubber asphalt and aggregate.

(4) CaCO_3_ clusters form a cluster structure with water molecules and reduce the water film thickness between rubber asphalt–aggregate. On the contrary, SiO_2_ clusters do not change the water film thickness between rubber asphalt–aggregate. The synergistic effect of the CaCO_3_ cluster structure and CaCO_3_ aggregate layer resulted in good adhesion characteristics and an anti-debonding effect of rubber/CaCO_3_ asphalt mastic–CaCO_3_ aggregate.

(5) While this study elucidates the fundamental mechanisms governing rubber asphalt–aggregate adhesion, several promising directions warrant further investigation: (1) extending the current framework to hybrid filler systems (e.g., CaCO_3_–SiO_2_ composites) to optimize synergistic effects; (2) evaluating long-term durability under coupled environmental–mechanical aging (UV/oxidation, freeze–thaw cycles, and traffic loading); (3) developing multiscale models bridging molecular interactions to macroscopic performance; and (4) exploring sustainable functionalization of fillers (e.g., nano-CaCO_3_ or bio-based modifiers) to enhance interfacial stability. Such advances would accelerate the design of high-performance rubber asphalt mixtures with tailored adhesion properties for diverse climatic and loading conditions.

## Figures and Tables

**Table 1:** Physical properties of the base asphalt.

Experiment	Requirements	Measured Value
Penetration (25 °C, 100 g, 5 s)/0.1 mm	80–100	89
Ductility 10 °C, 5 cm/min	≥100	>100
Softening point/°C	43–53	45.4
Dynamic viscosity 135 °C/Pa·s	≤3	2.7

**Table 2:** Physical properties of rubber powder.

Experiment	Requirements	Measured Value
Rubber hydrocarbon content (%)	≥42	58
Carbon black content (%)	≥22	31.58
Acetone extract (%)	6–16	8.35
Ash content (%)	≤8	5.13

**Table 3:** Physical properties of mineral powder.

Experiment	Requirements	Measured Value
Relative density (g/cm^3^)	≥2.50	2.73
Hydrophilicity coefficient (%)	<1	0.8

**Table 4:** XRF results of limestone.

Elemental Composition	CaO	SiO_2_	MgO	Al_2_O_3_	K_2_O	TiO	MnO	Fe_2_O_3_	RuO_2_
**Content/%**	52.63	26.56	19.36	0.00	0.00	0.00	0.01	1.10	0.34

**Table 5:** Test results of the surface energy parameters of the reagents.

Reagent Type	Surface Energy Parameters (mJ/m^2^)			
	$\gamma_{l}$	$\gamma_{l}^{d}$	$\gamma_{l}^{+}$	$\gamma_{l}^{-}$
**Distilled Water**	72.9	21.3	25.8	25.7
**Glycerin**	65	34	3.96	56.2
**Formamide**	56	39	2.27	39.5

**Table 6:** Test results of the surface energy parameters of the aggregate.

Aggregate	$\gamma_{s}$	$\gamma^{d}$	$\gamma^{p}$	$\gamma_{s}^{+}$	$\gamma_{s}^{-}$
**Limestone**	34.85	22.55	11.04	1.49	20.45

**Table 7:** Molecular numbers of the asphalt 12 components.

Type of Component		Molecular Formula	Number of Molecules
Saturate	SA-Ho	C_29_H_50_	5
	SA-Sq	C_30_H_62_	4
Aromatic	AR-Do	C_30_H_46_	18
	AR-Ph	C_35_H_44_	15
Resin	RE-Be	C_18_H_10_S_2_	15
	RE-Pv	C_36_H_57_N	4
	RE-Th	C_40_H_60_S	4
	RE-Qu	C_40_H_59_N	4
	RE-Tr	C_29_H_50_O	5
Asphaltene	AS-Ph	C_42_H_54_O	3
	AS-Py	C_66_H_81_N	2
	AS-Th	C_51_H_62_S	3

**Table 8:** Crystal parameters of SiO_2_ and CaCO_3._

Crystal Model	Chemical Formula	Lattice Parameters	Space Group	Group Name
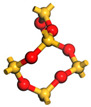	SiO_2_	a = b = 4.913 Å c = 5.4052 Å	α = β = 90° γ = 120°	P3121
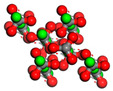	CaCO_3_	a = b = 4.99 Å c = 17.061 Å	α = β= 90° γ = 120°	R-3C

**Table 9:** Number of NR, CaCO_3_ cluster structures, and SiO_2_ cluster structures in the model of rubber asphalt (mastic).

Type of Asphalt Mastic	Number of Molecules		
	NR	CaCO_3_ Cluster	SiO_2_ Cluster
RA	4	-	-
RS1	4	-	1
RS2	4	-	2
RS3	4	-	3
RC1	4	1	-
RC2	4	2	-
RC3	4	3	-

## Data Availability

The original contributions presented in this study are included in the article. Further inquiries can be directed to the corresponding authors.
